# Association between Carotid Intima Media Thickness and Heart Rate Variability in Adults at Increased Cardiovascular Risk

**DOI:** 10.3389/fphys.2017.00248

**Published:** 2017-04-26

**Authors:** Valter L. Pereira, Mirela Dobre, Sandra G. dos Santos, Juliana S. Fuzatti, Carlos R. Oliveira, Luciana A. Campos, Andrei Brateanu, Ovidiu C. Baltatu

**Affiliations:** ^1^Center of Innovation, Technology and Education at Anhembi Morumbi University—Laureate International UniversitiesSao Jose dos Campos, Brazil; ^2^Heart Institute of Santa Casa Charity (IACOR)Fernandopolis, Brazil; ^3^Faculty of Medical Sciences, Brasil UniversityFernandópolis, Brasil; ^4^Division of Nephrology and Hypertension, University HospitalsCleveland, Ohio; ^5^Medicine Institute, Cleveland ClinicCleveland, Ohio

**Keywords:** carotid, atherosclerosis, heart rate/heart rate variability, cardiovagal, deep breathing test

## Abstract

**Background:** Atherosclerotic carotid intima-media thickness (IMT) may be associated with alterations in the sensitivity of carotid baroreceptors. The aim of this study was to investigate the association between carotid IMT and the autonomic modulation of heart rate variability (HRV).

**Methods:** A total of 101 subjects were enrolled in this prospective observational study. The carotid IMT was determined by duplex ultrasonography. The cardiac autonomic function was determined through HRV measures during the Deep Breathing Test. Linear regression models, adjusted for demographics, comorbidities, body mass index, waist-hip-ratio, and left ventricular ejection fraction were used to evaluate the association between HRV parameters and carotid IMT.

**Results:** Participants had a mean age of 60.4 ± 13.4 years and an estimated 10-year atherosclerotic cardiovascular disease (ASCVD) risk score (using the Pooled Cohort Equations) of 16.4 ± 17. The mean carotid media thickness was highest (0.90 ± 0.19 mm) in the first quartile of the standard deviation of all RR intervals (SDNN) (19.7 ± 5.1 ms) and progressively declined in each subsequent quartile to 0.82 ± 0.21 mm, 0.81 ± 0.16 mm, and 0.68 ± 0.19 in quartiles 2 (36.5 ± 5.9 ms), 3 (57.7 ± 6.2 ms) and 4 (100.9 ± 22.2 ms), respectively. In multivariable adjusted models, there was a statistical significant association between SDNN and carotid IMT (OR −0.002; 95%CI −0.003 to −0.001, *p* = 0.005). The same significant association was found between carotid IMT and other measures of HRV, including coefficient of variation of RR intervals (CV) and dispersion of points along the line of identity (SD2).

**Conclusions:** In a cohort of individuals at increased cardiovascular risk, carotid IMT as a marker of subclinical atherosclerosis was associated with alterations of HRV indicating an impaired cardiac autonomic control, independently of other cardiovascular risk factors.

## Introduction

Carotid atherosclerosis is characterized by increased intima-media thickness (IMT) and presence of atheromatous plaques in the arterial wall (Stein et al., 2008). The carotid IMT is a surrogate marker for systemic atherosclerosis and is associated with an increased risk of stroke and myocardial infarction (Bots et al., 1996, 1997). Increased carotid IMT is a risk factor for coronary and cerebrovascular events in patients without clinical cardiovascular disease (Arnold et al., 2005; Kuller et al., 2006). Duplex ultrasonography, a non-invasive method of measuring carotid IMT can be used to diagnose carotid artery atherosclerosis. However, current guidelines do not recommend using it for the routine screening of asymptomatic patients without clinical signs of or risk factors for atherosclerosis (Brott et al., 2011). Although routine screening for carotid artery stenosis is not recommended, the noninvasive carotid artery duplex ultrasonography in conjunction with other risk stratification markers may improve early diagnosis and prevention (Jonas et al., 2014; LeFevre and U. S. Preventive Services Task Force, 2014; Weyer and Davis, 2015).

Atherosclerosis of the carotid and aortic baroreceptors may be associated with cardiac autonomic dysfunction due to a decreased baroreflex sensitivity. Carotid atherosclerosis may induce these effects through both structural and biochemical mechanisms. Carotid atherosclerotic plaques are commonly found in the carotid sinus (Milei et al., 2009). Vlachakis et al. (1976) first suggested a role of atherosclerosis in diminishing baroreceptor sensitivity (Vlachakis et al., 1976). Several preclinical studies led to the hypothesis that structural and functional mechanisms in large arteries contribute to decreased baroreflex sensitivity and baroreceptor resetting in hypertension, atherosclerosis, and aging (Chapleau et al., 1995). Structural mechanisms that may contribute to the atherogenesis-induced reduction in baroreflex sensitivity include structural changes in the vessel wall, such as fibrosis with increased vascular stiffness and distensibility (Bonyhay et al., 1996). In the atherosclerotic carotid sinus, fibrotic autonomic ganglia and damaged nerve endings have been observed in both complicated and noncomplicated plaques (Milei et al., 2009). Since baroreceptors are located in the carotid bulb adventitia, decreased stretch caused by the presence of atherosclerotic plaques in this location may reduce the baroreflex sensitivity. Functional mechanisms may be paracrine through factors released by the vascular endothelium such as prostacyclin, nitric oxide and endothelin (Chapleau and Abboud, 1994) or involve the accumulation of oxygen-derived free radicals (Li et al., 1996). Therefore, carotid sinus baroreceptor dysfunction may occur, leading to alterations in the cardiac autonomic function.

Gianaros et al. associated intima–media thickness in the carotid bulb with reduced baroreflex sensitivity in patients enrolled in the REActivity and Cardiovascular risk Trial (REACT) to study preclinical atherosclerosis (Gianaros et al., 2002). Baroreflex sensitivity was found to be impaired in patients with bilateral carotid atherosclerosis (Nasr et al., 2005) or with echogenic plaques (Tsekouras et al., 2011).

Heart rate variability (HRV) analysis is a commonly used noninvasive method to measure alterations in baroreflex and autonomic tone (Campos et al., 2013; Costa et al., 2015; Miyabara et al., 2017). HRV measures are sensitive to age, gender, and cardiovascular diseases, such as diabetes, hypertension, obesity, dyslipidemia, and atherosclerosis (Campos et al., 2013; Jelinek et al., 2013). We hypothesized that indices of HRV analysis are independently associated with carotid atherosclerotic IMT in cohort with at least one cardiovascular disease risk factor. Since cardiovascular risk conditions and demographic factors could potentially affect HRV outcome variables (Campos et al., 2013), estimates for the association between HRV and carotid IMT were determined using linear multivariable modeling adjusting for these potential confounders.

## Methods

### Study design and setting

This was a prospective cross-sectional observational study. We enrolled 101 consecutive subjects who presented to the Cardiology outpatient clinic for a routine visit and agreed to have a noninvasive carotid artery duplex ultrasonography at Heart Institute of Santa Casa Charity (IACOR), Fernandopolis, Brazil, from June 2015 to February 2016. The study was approved by the Ethics Committee of Camilo Castelo Branco University in accordance with resolution 466/2012 and 340/2004 of the National Health Council (Ministry of Health) for research on human subjects (CAAE: 01328712.0.0000.5244, permit no. 18326). Written informed consent was obtained from all subjects.

### Inclusion and exclusion criteria

Asymptomatic patients aged 18 years and older with at least one cardiovascular risk factor, including hypertension, diabetes, obesity, smoking, hypercholesterolemia, without a history of transient ischemic attack, stroke, or other neurologic signs or symptoms were selected to participate in the study. Patients with symptomatic carotid artery stenosis were excluded if the patient had transient or permanent focal neurologic symptoms corresponding to the ipsilateral retina or the cerebral hemisphere (Lanzino et al., 2009). Also, patients with heart rhythm disorders, pacemaker, heart failure, ejection fraction less than 0.45, chronic renal failure on hemodialysis, severe liver failure, myocardial infarction, endarterectomy, carotid angioplasty, carotid dissection, and carotid occlusion were excluded. Drugs known to interfere with autonomic function, such as beta-blockers, calcium channel blockers, angiotensin-converting enzyme inhibitors, vasodilators, statins, neuroleptics and nasal vasoconstrictors, were discontinued for at least 24 h before the tests.

Clinical characteristics such as diabetes mellitus, hypertension, obesity, smoking, were documented together with the lipid profile (total cholesterol, triglycerides, LDL and HDL).

### Carotid atherosclerosis imaging

Carotid arteriosclerotic disease was evaluated through measuring the IMT. The subclinical vascular disease scanning protocol for evaluation of common carotid IMT and detection of carotid plaques was done according to the standardized protocol of the American Society of Echocardiography (Stein et al., 2008). Bilateral common carotid and internal carotid artery were evaluated using a high resolution ultrasound system (Philips, HD 11 XE, Andover MA, USA) equipped with 3–12 MHz linear transducer, SonoCT and XRES imaging technologies and QLAB quantification software. The images were documented through the media Digital Imaging and Communication in Medicine (DICOM). Ultrasound measurements were taken with the subjects in supine position. Carotid stenosis was investigated with an IMT automated measurement on the right and left common carotid arteries (Secil et al., 2005). The mean IMT value of the right and left common carotid arteries was calculated. Carotid IMT was measured on the posterior wall of the common carotid arteries 1–2 cm proximal to the carotid bifurcation and the proximal portion of the internal carotid (bulb) at a depth of 4 cm, by two-dimensional ultrasound (B-mode). Carotid IMT measurements were considered in longitudinal section, on the front or rear wall of the artery, the distance between two echogenic lines represented by the lumen-intima interface and media-adventitia of the arterial wall. The common mean of IMT measurements between the right and left sides (IMTm) was used. The regions containing plaques were excluded from the IMT measurements. Carotid plaque was considered at the bifurcation and proximal portion of the internal carotid artery and was defined as an IMT measurement greater than or equal to 1.5 mm. After the acquisition of the IMT values of each carotid artery, these were compared with the existing reference values, according to the normative table of the Multi-Ethnic Study of Atherosclerosis (MESA), used for both sexes, of White, black, Chinese or Hispanic ethnicity. In addition, left ventricular ejection fraction (LVEF) was measured with cardiac ultrasonography.

### Quantitative autonomic testing

Quantitative testing of the cardiac autonomic function was performed during deep breathing test (Low et al., 2013; Zeki Al Hazzouri et al., 2014). The HRV deep breathing test was measured in real-time and online with Faros wireless ECG monitor and HRV-scanner software (Mega Electronics, Finland) that uses a respiration pacer while measuring and analyzing the variability in the heart rate in response to deep breathing. The subjects were instructed to follow the respiration pacer bar, and to breathe as deeply as possible. The tests were performed with subjects in sitting position over six respiratory cycles and a respiratory rate of six breaths per minute. Cardiac autonomic function was evaluated measuring the linear and nonlinear HRV parameters: linear (standard deviation (SD) of normal R-R wave intervals [SDNN], square root of the mean of the sum of the squares of differences between adjacent normal R wave intervals [RMSSD], coefficient of variation [CV = SDNN/mean HR], the proportion derived by dividing NN50 by the total number of RR intervals [pNN50]), where NN50 represents the number of pairs of successive RRs that differ by more than 50 ms. and nonlinear Poincaré analysis (SD of the points perpendicular to the line-of-identity [SD1, ms]; SD along the line-of-identity [SD2, ms]).

### Statistical analysis

All data processing was performed while blinded to the level of deep breathing testing and analysis. The characteristics of study participants were depicted using standard descriptive statistics, overall and stratified by quartiles of SDNN. Chi-square (χ2) test of independence for categorical (nominal) variables and Analysis of variance (ANOVA) test for continuous variables were used to analyze the covariates of interest and their association with SDNN and other measures of HRV (RMSSD, CV, PNN50, SD1, and SD2).

Multivariable linear regression models were used to assess the association between heart rate variability parameters from the deep breathing test and mean carotid IMT.

The goodness of curve fit was assessed with the *F*-test.

The models were adjusted for relevant confounding variables, including demographics (age, sex, race/ethnicity), traditional CVD risk factors (hypertension, diabetes, smoking, total cholesterol, and high density lipoprotein) grouped into atherosclerotic cardiovascular disease (ASCVD) risk score, body mass index (BMI), waist-hip-ratio (WHR), and left ventricular ejection fraction (LVEF). Sequential multivariable models for each outcome were created based on our assessment of the covariates likelihood of being a confounder in the relationship between heart rate variability and subclinical carotid disease. Residual plots were used to confirm model assumptions.

All statistical tests were 2 sided, and *P* < 0.05 was considered significant. IBM Corp (2013) SPSS Statistics for Windows, Version 22.0, Armonk, NY was used for analyses.

## Results

### Patient characteristics

A total of 101 study participants met the eligibility criteria and performed the deep breathing test. The mean age was 60.4 ± 13.4(SD) years, and 46% were men. Characteristics of study participants distributed according to quartiles of one of the HRV parameters (standard deviation of all RR intervals (SDNN) from the deep breathing test) are summarized in Table 1. Patients in the lowest vs. highest quartile of SDNN (reduced vs. high heart rate variability) were older (70.6 ± 10.9 vs. 49.9 ± 13.2 years), had higher supine systolic blood pressure (145.7 ± 18.8 vs. 129.0 ± 15.1 mmHg), and ASCVD risk score (27.3 ± 17.2 vs. 8.2 ± 10.7), respectively.

**Table 1:** **Characteristics of study participants by quartile of heart rate variability (SDNN) from the Deep Breathing Test**.

**Characteristics**	**All participants *N* = 101**	**Quartiles of SDNN**				***p*-value**
		***N* = 25**	***N* = 26**	***N* = 25**	***N* = 25**	
SDNN (ms)	53.5 ± 32.7	19.7 ± 5.1	36.5 ± 5.9	57.7 ± 6.2	100.9 ± 22.2	
Age (years)	60.4 ± 13.4	70.6 ± 10.9	61.6 ± 11.7	59.4 ± 9.3	49.9 ± 13.2	<0.001
Men, n %	46 (45.5)	7 (28.0)	12 (46.2)	13 (52.0)	14 (56.0)	0.20
Race, n %						0.80
White	96 (95.0)	24 (96.0)	25 (96.2)	24 (96.0)	23 (92.0)	
Black	4 (4.0)	1 (4.0)	1 (3.8)	1 (4.0)	1 (4.0)	
Other	1 (1.0)	0 (0.0)	0 (0.0)	0 (0.0)	1 (4.0)	
BMI (kg/m^2^)	27.7 ± 4.4	28.4 ± 4.9	28.3 ± 4.3	27.3 ± 4.5	27.0 ± 3.9	0.58
WHR	0.9 ± 0.1	0.9 ± 0.1	0.9 ± 0.1	0.9 ± 0.1	0.9 ± 0.1	0.62
Smoking	16 (15.8)	4 (16.0)	6 (23.1)	4 (16.0)	2 (8.0)	0.54
HTN	48 (47.5)	16 (64.0)	12 (48.0)	12 (48.0)	8 (32.0)	0.16
DM	17 (16.8)	6 (24.0)	4 (15.4)	5 (20.0)	2 (8.0)	0.47
SBP supine (mmHg)	137.0 ± 17.6	145.7 ± 18.8	137.8 ± 19.2	135.5 ± 13.3	129.0 ± 15.1	0.007
SBP standing (mmHg)	129.1 ± 17.0	135.2 ± 16.8	130.0 ± 20.1	127.5 ± 15.1	123.5 ± 14.1	0.09
DBP (mmHg)	78.4 ± 10.5	79.3 ± 10.7	78.3 ± 13.0	78.3 ± 9.7	78.0 ± 8.2	0.97
LVEF (%)	67.8 ± 4.4	69.5 ± 5.4	65.9 ± 3.9	68.7 ± 3.9	67.1 ± 3.3	0.01
TC (mg/dl)	191.2 ± 39.4	190.7 ± 41.3	191.4 ± 38.5	189.4 ± 39.9	193.3 ± 40.2	0.99
HDL (mg/dl)	46.3 ± 9.6	47.3 ± 10.5	43.1 ± 8.1	48.0 ± 10.2	47.0 ± 9.0	0.25
LDL (mg/dl)	113.0 ± 35.9	110.5 ± 35.3	113.6 ± 33.7	108.8 ± 38.6	119.2 ± 37.2	0.75
ASCVD risk score	16.4 ± 17.5	27.3 ± 17.2	19.0 ± 22.3	10.9 ± 10.3	8.2 ± 10.7	<0.001
IMT (mm)	0.80 ± 0.20	0.90 ± 0.19	0.82 ± 0.21	0.81 ± 0.16	0.68 ± 0.19	0.001

*Numbers represent N (%) or Mean ± SD. SDNN, standard deviation of all RR intervals; BMI, body mass index; WHR, waist-hip-ratio; HTN, hypertension; DM, diabetes mellitus; SBP, systolic blood pressure; DBP, diastolic blood pressure; LVEF, left ventricular ejection fraction; TC, total cholesterol; HDL, high-density lipoprotein; LDL, low-density lipoprotein; ASCVD, atherosclerotic cardiovascular disease, IMT, carotid intima media thickness*.

The participants had a carotid intima media thickness mean 0.80 ± 0.20 mm with a median [IQR] 0.80 [0.65–0.93] mm. The characteristics of HRV parameters are depicted in Table 2.

**Table 2:** **Characteristics of heart rate variability parameters from deep breathing test (***N*** = 101)**.

**HRV Parameters**	**Median [IQR]**
SDNN (ms)	47.3 [26.5–69.4]
CV	5.3 [3.1–7.7]
RMSSD (ms)	27.5 [17.5–51.1]
PNN50	7.3 [1.2–26.7]
SD1	19.4 [12.4–36.2]
SD2	63.8 [34.7–90.4]

*SDNN, standard deviation of all normal RR intervals; CV, coefficient of variation of RR intervals; RMSSD, root-mean square of differences between adjacent normal RR intervals; PNN50, the proportion of NN50 divided by total number of RRs, where NN50 represents the number of pairs of successive RRs that differ by more than 50 ms.; SD1, dispersion of points perpendicular to the axis of line-of-identity; SD2, dispersion of points along the line of identity; SD1/SD2, ratio of SD1 to SD2*.

### Association between heart rate variability and intima media thickness

**Figure 1:**
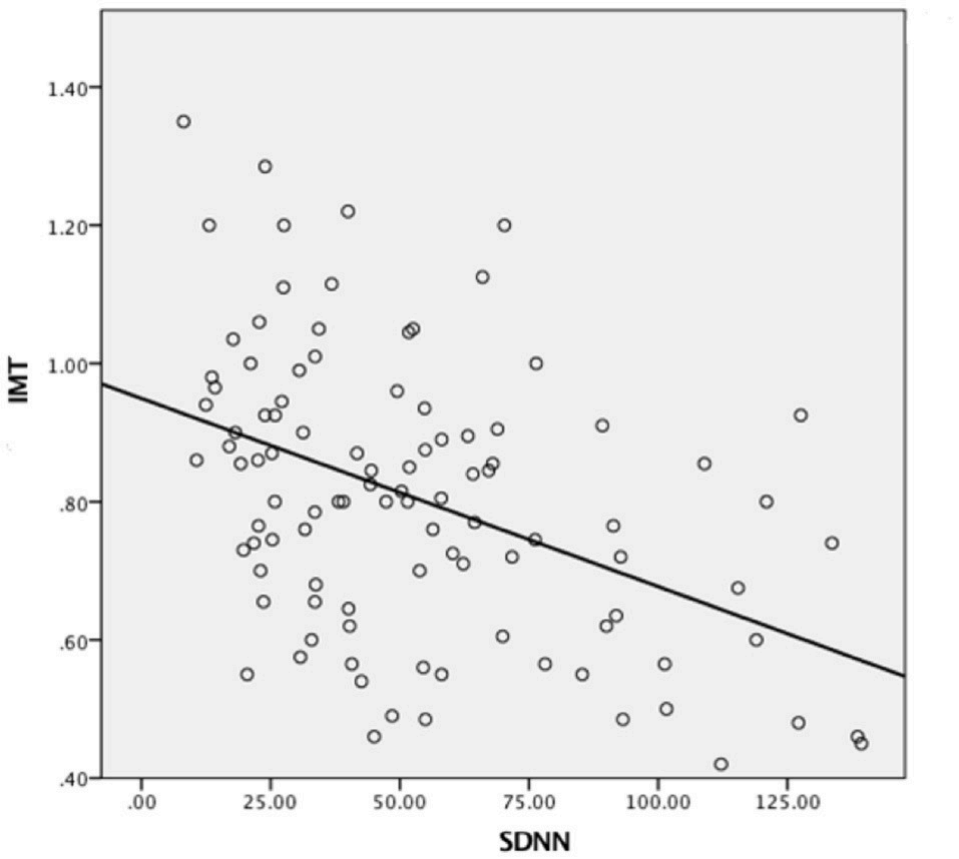
**Scatterplot of intima media thickness by SDNN from deep breathing test**.

**Figure 2:**
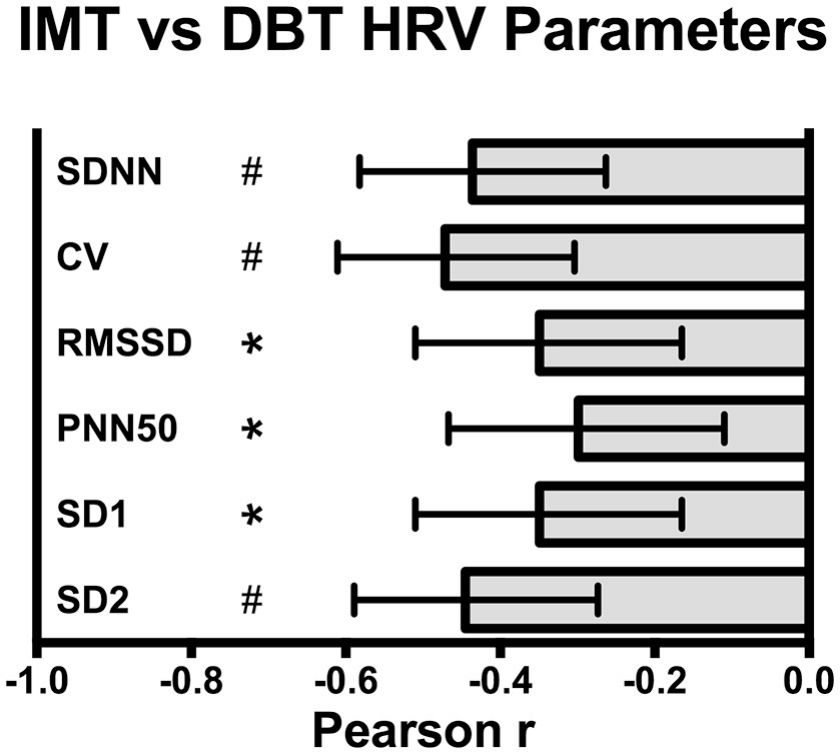
**Pearson correlation between carotid intima media thickness (IMT) and deep breathing test (DBT) heart rate variability (HRV) parameters: SDNN, standard deviation of all RR intervals, CV, coefficient of variation of RR intervals, RMSSD, root-mean square of differences between adjacent normal RR intervals, SD1, dispersion of points perpendicular to the axis of line-of-identity, SD2, dispersion of points along the line of identity**. Values are Pearson r and 95% CI, ^*^p < 0.005, ^#^*p* < 0.0001.

Scatterplot distribution of IMT by SDNN from the deep breathing test is shown in Figure 1. Similar distribution was observed for IMT by CV and SD2 (data not shown). The Pearson correlation between carotid IMT and HRV parameters from the deep breathing test showed a significant negative correlation: −0.44 [95%CI: −0.58 to −0.26], −0.47 [95%CI: −0.61 to −0.3], and −0.44 [95%CI: −0.59 to −0.27] for SDNN, CV and SD2, respectively (Figure 2).

The median carotid media thickness was highest (0.90 ± 0.19 mm) in the first quartile of SDNN (19.7 ± 5.1 ms) and progressively declined in each subsequent quartile to 0.82 ± 0.21 mm, 0.81 ± 0.16 mm, and 0.68 ± 0.19 in quartiles 2 (36.5 ± 5.9 ms), 3 (57.7 ± 6.2 ms) and 4 (100.9 ± 22.2 ms), respectively (*p* = 0.001 between groups). In multivariable adjusted models, there was a statistical significant association between SDNN and carotid IMT. Each 1 ms increase in SDNN is associated with a reduction by 2 μm in carotid IMT (OR −0.002; 95%CI −0.003 to −0.001, *p* = 0.005). The same significant association was found between carotid IMT and other measures of HRV: coefficient of variation (CV) of RR intervals (OR −0.016; 95%CI −0.026 to −0.006, *p* = 0.002), root-mean square of differences between adjacent normal RR intervals (RMSSD) (OR −0.002; 95%CI −0.003 to −0.000, *p* = 0.03), dispersion of points perpendicular to the axis of line-of-identity (SD1) (OR −0.002; 95%CI −0.005 to −0.000, *p* = 0.03), and dispersion of points along the line of identity (SD2) (OR −0.001; 95%CI −0.002 to −0.000, *p* = 0.004) (Table 3).

**Table 3:** **Association between Heart Rate Variability (HRV) Parameters from the Deep Breathing Test and mean Carotid Artery Intimal-Media Thickness (CIMT)**.

**HRV parameters**	**CIMT (mm)**					
	**Unadjusted model**			Adjusted model^*^		
	**Odds Ratio**	**95% CI**	***P*-value**	**Odds Ratio**	**95% CI**	***P*-value**
SDNN (ms)	−0.003	−0.004 to −0.002	<0.001	−0.002	−0.003 to −0.001	0.005
CV	−0.025	−0.035 to −0.016	<0.001	−0.016	−0.026 to −0.006	0.002
RMSSD (ms)	−0.003	−0.005 to −0.001	<0.001	−0.002	−0.003 to −0.000	0.03
PNN50	−0.004	−0.006 to −0.001	0.002	−0.002	−0.004 to −0.000	0.1
SD1	−0.004	−0.006 to −0.002	<0.001	−0.002	−0.005 to −0.000	0.03
SD2	−0.002	−0.003 to −0.001	<0.001	−0.001	−0.002 to 0.000	0.004

* *All models adjusted for atherosclerotic cardiovascular disease (ASCVD) risk score (to include age, gender, race, HDL and total cholesterol, diabetes, systolic blood pressure, hypertension treatment and smoking), body mass index (BMI), waist-hip-ratio (WHR), left ventricular ejection fraction (LVEF); SDNN, standard deviation of all normal RR intervals; CV, coefficient of variation of RR intervals; RMSSD, root-mean square of differences between adjacent normal RR intervals; PNN50, the proportion of NN50 divided by total number of RRs, where NN50 represents the number of pairs of successive RRs that differ by more than 50 ms.; SD1, dispersion of points perpendicular to the axis of line-of-identity; SD2, dispersion of points along the line of identity; SD1/SD2, ratio of SD1 to SD2*.

## Discussion

In this cohort of individuals with subclinical carotid atherosclerosis and at increased cardiovascular risk, carotid IMT as a marker of subclinical atherosclerosis was associated with alterations of HRV indicating an impaired cardiac autonomic control. This association was found to be independent of atherosclerotic cardiovascular disease (ASCVD) risk score (to include age, gender, race, HDL, and total cholesterol, diabetes, systolic blood pressure, hypertension treatment and smoking), body mass index (BMI), waist-hip-ratio (WHR), left ventricular ejection fraction (LVEF). By demonstrating a significant inverse relationship between carotid IMT and HRV, our results are in line with the hypothesis that carotid atherosclerotic lesions are associated with impaired ANS function.

We previously investigated the relationship between carotid IMT and cardiovagal dysfunction assessed through frequency (Pereira et al., 2013) and time-domain (Pereira et al., 2015) HRV measures of 20-min office ECG measurement in patients with subclinical carotid atherosclerosis. Time-domain indices of HRV had the strongest inverse correlation with carotid IMT. Other groups have found a significant correlation between carotid IMT and baroreflex in hypertensives possibly due to aging (Labrova et al., 2005). Also, an increased carotid IMT was associated with a significant decrease in baroreflex sensitivity in prehypertensives and hypertensives (Celovska et al., 2017). In type 2 diabetic patients, cardiovascular autonomic neuropathy was associated with carotid atherosclerosis (Meyer et al., 2004; Gottsater et al., 2005). Moreover, decreased heart rate variability as a measure of cardiac autonomic neuropathy may predict the progression of carotid atherosclerosis in type 2 diabetes (Gottsater et al., 2006; Miyamoto et al., 2010). In subjects with preclinical atherosclerosis, a higher task-induced cardiac autonomic reactivity was associated with lower carotid IMT even after adjusting for cardiovascular risk factors (Heponiemi et al., 2007; Chumaeva et al., 2009). The Hyogo Sleep Cardio-Autonomic Atherosclerosis (HSCAA) Study identified HRV parameters associated significantly with carotid plaque, independent of sleep condition (Kadoya et al., 2015).

Our study indicates that carotid IMT as marker of subclinical atherosclerosis is associated with impaired cardiac autonomic control as represented by a decrease in HRV indices. Moreover, the relationship between deep breathing HRV and subclinical carotid disease was maintained after adjustment for relevant confounding variables, including traditional CVD risk factors (age, sex, race, hypertension, diabetes, smoking, total cholesterol, and high density lipoprotein) grouped into the atherosclerotic cardiovascular disease (ASCVD) risk score, body mass index (BMI), waist-hip-ratio (WHR), and left ventricular ejection fraction.

HRV has been studied in conjunction with clinical autonomic tests, of which the most used are the Valsalva maneuver, head-up tilt, and deep breathing tests (1996; Novak, 2011; Freeman and Chapleau, 2013). Of these, we selected the autonomic deep breath testing, which has been used as a sensitive and reliable clinical test for the early detection of cardiac autonomic dysfunction in various diseases (Olney, 1998; Hilz and Dutsch, 2006; Shields, 2009). Deep breathing test appears to have the greatest specificity (~80%) (Tesfaye et al., 2010). This test is based on the observation first noted by Karl Ludwig in 1847 that heart rate increases with inspiration and decreases with expiration (Melcher, 1976; Shields, 2009). In our study, we employed user-friendly portable equipment for HRV deep breathing test that could be used both in the office and under ambulatory conditions. The test has good sensitivity, specificity and reproducibility (it has reproducibility required for use in clinical studies), is noninvasive, safe, well standardized and easy to perform. In this study, we observed that time-domain measures of the HRV deep breathing test had again a strong negative correlation with the carotid IMT.

This study has several strengths, including concurrent assessment of bilateral common carotid and internal carotid artery media thickness, in conjunction with several indices related to cardiac autonomic function during deep breathing test, including SDNN, RMSSD, CV, pNN50, SD1, and SD2, and along with several cardiovascular risk factors. In the present study, multivariable regression analysis revealed a significant inverse association between carotid IMT and HRV that is independent of several cardiovascular risk factors. The cross-sectional design of our study was also appropriate to examine several cardiovascular risk factors related with HRV. To quantify autonomic dysfunction, we utilized comprehensive, portable and easy to use heart rate variability analyzing system that can be easily carried out in any patient setting. HRV deep breathing test is a fast and convenient method to quantify autonomic dysfunction. HRV measures have been suggested for initial screening of cardiovascular risk as it is noninvasive, economical and easy to use in clinical practice (Jelinek et al., 2013). We suggest further investigation of this association of HRV measures of deep breathing test to carotid IMT in larger cohort, case-control and longitudinal studies in order to certify HRV measures of deep breathing test as initial screening tool for adults with subclinical carotid disease and at increased cardiovascular risk.

This study has several limitations. First, this is a cross-sectional analysis and causality cannot be inferred. Second limitation relates to selection bias, given the nature of high risk patients referred directly from the Cardiology clinic. Furthermore, the results are only applicable to adults with at least one cardiovascular risk factor. Finally, these analyses are based on a sample of patients referred for single measurement of carotid IMT; whether changes in IMT over time add incremental predictive value to the development of cardiac autonomic dysfunction will need further study.

In summary, our study indicates that subclinical carotid atherosclerosis is associated with impairment in the cardiac autonomic control in individuals with at least one cardiovascular disease risk factor, regardless of the confounding effects of cardiovascular risk factors.

## Author contributions

Study conception and design: OB, VP, and LC. Performed the study: VP, Sd and JF. Analyzed de data: MD, AB, and VP. Drafting of manuscript: OB, LC. Critical revision: MD, AB, VP, and CO.

### Conflict of interest statement

The authors declare that the research was conducted in the absence of any commercial or financial relationships that could be construed as a potential conflict of interest.
